# Using Dimensionality Reduction Techniques for Refining Passive Indoor Positioning Systems Based on Radio Fingerprinting

**DOI:** 10.3390/s17040871

**Published:** 2017-04-15

**Authors:** Pedro E. Lopez-de-Teruel, Oscar Canovas, Felix J. Garcia

**Affiliations:** Department of Computer Engineering, University of Murcia, 30100 Murcia, Spain; pedroe@um.es (P.E.L.-d.-T.); fgarcia@um.es (F.J.G.)

**Keywords:** dimensionality reduction, fingerprinting, RSSI, passive localization, visualization

## Abstract

Indoor positioning methods based on fingerprinting and radio signals rely on the quality of the radio map. For example, for room-level classification purposes, it is required that the signal observations related to each room exhibit significant differences in their RSSI values. However, it is difficult to verify and visualize that separability since radio maps are constituted by multi-dimensional observations whose dimension is directly related to the number of access points or monitors being employed for localization purposes. In this paper, we propose a refinement cycle for passive indoor positioning systems, which is based on dimensionality reduction techniques, to evaluate the quality of a radio map. By means of these techniques and our own data representation, we have defined two different visualization methods to obtain graphical information about the quality of a particular radio map in terms of overlapping areas and outliers. That information will be useful to determine whether new monitors are required or some existing ones should be moved. We have performed an exhaustive experimental analysis based on a variety of different scenarios, some deployed by our own research group and others corresponding to a well-known existing dataset widely analyzed by the community, in order to validate our proposal. As we will show, among the different combinations of data representation methods and dimensionality reduction techniques that we discuss, we have found that there are some specific configurations that are more useful in order to perform the refinement process.

## 1. Introduction

Indoor positioning services provide specific locations of mobile devices, and they are an important building block for ubiquitous computing services. In recent years, several solutions have been proposed for the indoor positioning problem [1], most of them based on 802.11 radio signals due to the high density of access points in urban environments. Some active approaches assume that a specific software component is running on the mobile devices in order to collect the radio signals. Others perform passive localization, which makes use only of monitors collecting the radio signals generated by mobile devices in their usual operation.

Our recent work [2] is more focused on the latter type of positioning systems, since they do not require the explicit collaboration of the users nor the installation of special purpose applications on their mobile devices. Generally speaking, users are reluctant to install apps that are battery consuming, and additionally, there are serious limitations to obtain RSSI (Received Signal Strength Indicator) information from some operating systems. Moreover, the proliferation of smartphones and tablets is enabling new possibilities in order to infer information about the behavior and the activities of the users carrying those devices. Passive systems are suitable for that kind of activity recognition for large communities of users since they impose less requirements on the users’ side.

Independent of the active or passive approach, fingerprinting relies on a training phase, which requires gathering signal strength observations from particular emitters at reference spots in the operation area and storing them together with their physical coordinates in a radio map. Those training observations are multi-dimensional according to the number of emitters, and it is desirable that different areas of interest exhibit different radio patterns in order to minimize the estimation error during the localization phase. The distribution of those emitters (access points for active systems or monitors acting like access points during the training phase for passive systems) affects drastically the expected performance of a particular positioning system. Therefore, it is desirable to have a method able to provide rapid and intuitive feedback about the suitability of that distribution in terms of the expected accuracy error, which is directly related to the degree of separability between the observations composing the radio map. However, due to the multi-dimensional nature of the data, it is not straightforward to visualize that separability.

In this paper, we make use of several dimensionality reduction techniques to address the visualization problem. This proposal was schematically introduced in [3], but here, we include an extensive analysis of several datasets and additional techniques. We can find in the literature some works making use of this kind of technique in the field of indoor positioning. Fang et al. [4] show that the size of training samples can be reduced in an uncorrelated space, and they make use, for example, of PCA (Principal Component Analysis) [5] to improve the positioning accuracy. Ma et al. [6] employ local discriminant embedding [7], a variant of the more classical and widely known LDA technique [8], which is itself an improvement of PCA when treating with supervised data, i.e., when fingerprinting data are labeled by zones, to reduce both the dimensionality of the training radio map and the fingerprints obtained during the on-line phase. These works are focused on the use of dimensionality reduction to improve the accuracy and efficiency of the estimations. Alternatively, our proposal is instead more aligned with the one of Lemelson et al. [9] in the sense that, just as in their work, we will also obtain clusters of signals from the radio map that exhibit similar behavior. In our case, nevertheless, that clustering will be used to provide useful visual feedback information about the suitability of the radio map given the current monitor positions in relation to the desired zone partition for classification, rather than to estimate a positioning error measure. One of the most promising techniques for this purpose is the recently proposed t-SNE (t-distributed Stochastic Neighbor Embedding) [10]. This technique has numerous advantages with respect to more classical approaches to dimensionality reduction, such as the aforementioned PCA and LDA, this being especially true in the specific case of indoor positioning, as we will show. To the best of our knowledge, this is the first work in which this technique is applied to this field.

Visualization can also be used to compare different methods of fingerprinting data representation and their distance metrics. As we referred to above, passive localization has to deal with heterogeneous devices generating signals with different RSSI and temporal patterns. It is usually impractical [11] to adopt calibration methods in order to adjust the signal measurements to some reference values. Consequently, representation methods that tolerate device diversity are necessary. A possible solution is to base fingerprinting data representation on the order relationship between RSSI values, instead of using absolute (raw) signal strength values, an approach followed by systems like FreeLoc [12]. In order to solve the heterogeneity problem, while still allowing the use of general dimensionality reduction techniques, we propose our own order-based vector representation, called ternary vectors, which, as we will demonstrate, shows important advantages in the specific case of passive localization.

Finally, cluster visualization can be especially intuitive when combined with geometric maps. In this paper, we also present two novel visualization methods that generate a graphical layer to be displayed on top of the map of the target scenario. By combining the ternary vector representation with our method of choice, t-SNE, we propose two map visualization tools, one based on arrows and another one based on colors, which clearly simplify the process of identifying conflict zones that could require additional monitors or a better distribution of the existing ones, as well as quickly detecting observation outliers caused by the noisy nature of 802.11 signals.

In order to evaluate our proposals, we have performed an exhaustive validation process using data from three datasets based on two different radio technologies, 802.11 and Bluetooth Low Energy (BLE). One of the 802.11 datasets is derived from an existing indoor positioning system deployed in our own institution, while the other is the UJIindoorlocdataset [13], a public multi-building and multi-floor localization database based on WLAN fingerprinting, which is being widely used by the research community for testing purposes about localization performance [14]. Finally, a BLE dataset is also deployed in our university that will be useful to illustrate that our proposal works well with different radio technologies, not only for 802.11-based systems.

The main contributions of this work can therefore be summarized as follows:

- A specific deployment cycle to refine the training radio maps required for passive indoor positioning systems, based on the use of dimensionality reduction techniques.
- An exhaustive experimental analysis including several datasets in order to determine the best techniques for both data representation and dimensionality reduction. We found that the t-SNE technique, not applied until now to the field of fingerprinting-based indoor localization, combined with the novel ternary vector representation, outperforms more classical approaches like PCA and LDA based on direct RSSI measures.
- Two different visualization methods for quick identification of overlapping areas and outliers directly over the scenario map.

The rest of the article is structured as follows. Section 2 presents an overview of the deployment cycle designed to refine passive localization systems. The different proposed data representations and associated metrics are presented in Section 3. Then, an overview of the different dimensionality reduction techniques that we have employed for our analysis is presented in Section 4. Section 5 contains a thorough experimental evaluation of the different dimensionality reduction techniques to illustrate their suitability to know the degree of separability of the training observations corresponding to different types of scenarios. The visualization methods that we propose to display separability on top of geometric maps are presented in Section 6. Finally, conclusions and future work are drawn in Section 7.

## 2. Overview

Our work is mostly motivated by the specific peculiarities of passive indoor positioning. The following set of features intrinsically characterize passive localization systems, in contrast with active ones:

- There is no need for special software installed on the mobile devices to be tracked.
- They require the deployment of special purpose devices, usually called monitors, whose number and specific positions are decided by the system designers.
- Estimations and other calculations are performed by an external server, instead of in the mobile devices themselves.
- Mobile devices are assumed to be heterogeneous, so an uncalibrated approach is required.
- Traffic patterns are unpredictable since there is no (or minimal) control over the mobile devices.
- The granularity of the localization tends to be coarser, based on zone classification, rather than exact position regression.

**Figure 1:**
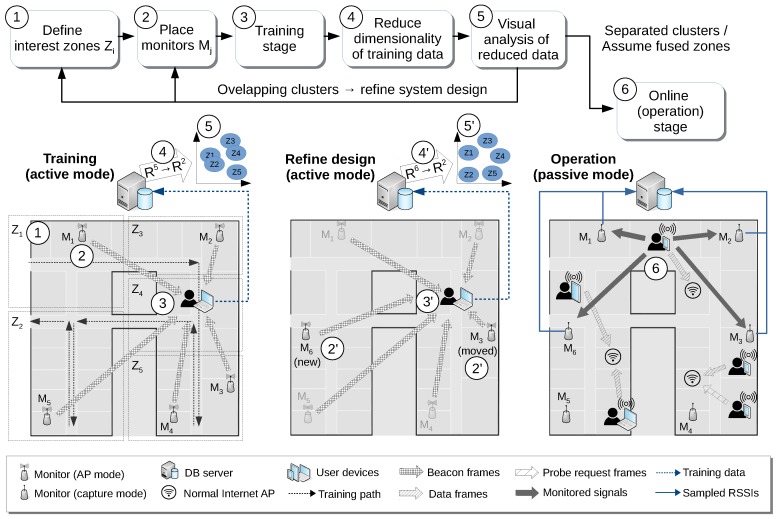
Overview of the refinement process.

Figure 1 shows an overview of our proposal for the deployment cycle of a typical passive localization system. First of all, the system designers must decide on a set of interest zones $Z_{i}$ in the target scenario (1), as well as the number of monitors $M_{j}$ to deploy and their respective initial positions (2). In a broad sense, a monitor is any hardware element running software able to capture wireless (802.11 or BLE) traffic and export the relevant information of this captured data to a central server. Typical cases of monitors could be (a) a wireless router with adapted firmware, (b) a dedicated WiFi adapter in a standard PC or (c) a small single board computer, for example a Raspberry Pi, endowed with WiFi/BLE interface, among other possibilities. The only requirement is that they must be able to be configured to capture wireless traffic in promiscuous mode, because monitors rely only on capturing the frames transmitted by the mobile devices as part of their usual connections or active scanning periods (in this work, we do not consider prompting techniques [15] to increase the number of packets received from them). The required density of monitors can be relatively low for many localization scenarios, but this of course highly depends on both the desired accuracy (i.e., the spatial granularity of the localization system) and the physical characteristics of the environment itself.

Then, as in any fingerprinting-based technique, a training phase, which involves a manual site survey process, is performed (3), where an operator carrying a device running specific software follows a given walking path. In our case, we use an application that provides continuous visual feedback about the required walking path for the site survey, thus providing exact geopositioning for the training data. During this phase, monitors, instead of capturing traffic, are configured to broadcast beacon or advertising frames (i.e., we set them in access point mode, for 802.11, or advertising mode, for the case of BLE). The RSSIs observed by the training device for those frames are then recorded by the application and used to save the corresponding geotagged observations. Each of these observations is thus formed by a vector of RSSI measures of dimension equal to the number of monitors, plus a ground truth (x,y) position in a coordinate system locally defined in the scenario. Using this position, the specific zone of each vector can also be easily determined.

This is where the core of our proposal of dimensionality reduction comes into play. To know whether the current position of the monitors allows for correct classification of users’ location according to the desired zone partition, we propose to reduce the (usually high) dimensionality of the input training vectors (4) in order to appreciate visually if the training samples, grouped by zones, show clear overlapping areas (5). In that case, the system designers will have a valuable clue of where the specific conflict zones are and thus might decide to move some of the monitors, or even to add new ones, in order to alleviate the overlapping problem (2’). Alternatively, they could just redefine the interest zones, fusing the ones that clearly overlap given the current position of the monitors. In any case, if any change in the number and/or position of the monitors is performed, a new training process will be needed (3’), from which a new dimensionality reduction (4’) and corresponding cluster visualization (5’) will be obtained.

Once system designers have decided that the current monitor positions and distribution of zones are adequate, and using the corresponding training data as the final fingerprint map, monitors can be switched to capture mode, and the system can start its operation (6). We assume that the user mobile devices (smartphones, tablets, laptops, etc.) being monitored will show a wide variety of hardware, WiFi interfaces, antennas, operating systems and the like. Moreover, some of them could be connected to local available Internet access points, thus generating normal data frames traffic, while others could be not currently connected, and thus just generating sporadic probe frames in order to request information from available access points. Consequently, they will produce signals with very different strength and temporal patterns. Note also that training vectors are based on the RSSI of frames emitted from our monitors and captured by the training device, while in the operational phase, just the opposite occurs, that is RSSIs are obtained for frames emitted by user devices and measured by the monitoring elements. Though the respective RSSIs will be clearly correlated, as determined by the device-monitor distance, in general, these measures will not have to be exactly the same. This asymmetry adds another source of heterogeneity to the involved RSSIs’ fingerprints. For all of these reasons, heterogeneity has to be addressed in passive localization systems, and in our proposal, this important issue will imply some design decisions regarding data representation, which will be explained in the next section. This heterogeneity support will also be required if we want to employ some kind of incremental or organic approach [16,17] to update the radio map in a periodic basis.

## 3. Data Representation and Distance Metrics

### 3.1. Data Representation

As was stated in the Introduction, our proposal will be aware of device heterogeneity by using an alternative data representation method, which is mainly based on the order relationship information between RSSI values, discarding the absolute values that require the adoption of calibration methods. In this section, we discuss that representation, which we call ternary vectors, by comparing it with the more classical RSSI-based representation, here called raw vectors.

#### 3.1.1. Raw Vectors

Classical representation for fingerprinting data makes use of raw RSSI measures, building vectors $r=\left(r_{1},\ldots ,r_{M}\right)\in R^{M}$ for every observation obtained after an active scanning period performed by the training device (thus the name raw vectors). Here, *M* stands for the number of monitors for each particular scenario. Despite the fact that it depends on the particular device being used for training purposes, usually $r_{i}$ refers to the maximum RSSI value (in dBm) observed for an 802.11 or BLE beacon frame transmitted from monitor $m_{i}$ in the corresponding scanning period (which also varies from one device to another). If any $r_{i}$ value is unavailable (due to the fact that the corresponding beacon frame was not received from the monitor), a minimum value of −100 dBm is assigned to it, in order to get a completely-defined vector.

#### 3.1.2. Ternary Vectors

The idea behind the ternary vectors $t=(t_{1},\ldots ,t_{M^{\prime }})$ is to represent just the magnitude relationship between the RSSI measurements of a raw vector, thus discarding the specific $r_{i}$ values, which might not be very useful due to the already discussed issue of device heterogeneity. These alternative vectors will always be longer than the corresponding raw vectors (i.e., $M^{\prime }>>M$), as we will explain shortly, and the name of “ternary” comes from the fact that, whatever the resulting dimensionality $M^{\prime }$, each individual component of each vector will always take one of the three values {−1,0,+1}, as opposed to the continuous RSSIs scalar values measured in dBm of raw vectors components. As long as the relative order of the signal strengths obtained from the different monitors is maintained, fluctuations in the RSSI values will not modify these ternary values, which is very suitable for dealing with heterogeneous devices.

In order to achieve that, each t vector is built from r taking all of the M′=(M2)=M*(M−1)2 combinations of the *M* monitors by pairs and using a tolerance parameter δ, in a way that two components $r_{i}$ and $r_{j}$ are considered to be equal when $|r_{i}-r_{j}|<=\delta$ dBm. The δ parameter serves to enforce a significant difference between RSSI values for it to be considered relevant, just as in the FreeLoc system [12]. Reasonable values for this parameter could lie in the interval 0≤δ≤5 dBm. Each of these pairwise comparisons can then give rise to three different values +1, −1 or 0 (thus the name of ternary vectors): ∀c∈{(i,j)|i,j∈{1,…,M},i<j}, we define $t_{c}=+1$ if $r_{i}-r_{j}>=\delta$ (or simply, we did not receive any frame from $m_{j}$); $t_{c}=-1$ if $r_{i}-r_{j}<=-\delta$ (or we did not receive any frame from $m_{i}$); and $t_{c}=0$ when $|r_{i}-r_{j}|<\delta$ or neither $m_{i}$ nor $m_{j}$ were received.

As an example, using the following input raw vector for a theoretical scenario of four monitors, $r=\left(r_{1},\ldots ,r_{4}\right)=\left(-60,-80,-50,-62\right)$, the corresponding output vector $t=(t_{1},\ldots ,t_{6})$ would be (+1,−1,+1,−1,−1,+1) for δ=0, or (+1,−1,0,−1,−1,+1) for δ=5, where the positions 1…6 represent the (42) possible pair comparisons {(1,2),(1,3),(1,4),(2,3),(2,4),(3,4)} for each ternary vector, in that order.

### 3.2. Distance Metrics

As we will see in the following section, dimensionality reduction techniques require distance metrics to accomplish their task. Most of these techniques are based on the standard euclidean distance, but others can make use of alternative metrics. In this section, we briefly introduce the two different metrics that we have tested.

1. Euclidean distance will be used both for raw vectors and for ternary vectors in those cases where the dimensionality reduction technique is not able to work with a custom metric. Given two vectors (raw or ternary) $v_{a}$ and $v_{b}$, it is defined as:
  (1) $$EU_{v_{a},v_{b}}=∥v_{a}-v_{b}∥$$
2. FreeLoc distance is only used for ternary vectors and for those techniques that support custom metrics. Given two ternary vectors $t_{a}$ and $t_{b}$, it is defined as:
  (2) $$FL_{t_{a},t_{b}}=K-\left|C_{t_{a},t_{b}}\right|$$
  Here, $C_{t_{a},t_{b}}$ represents the set of pairs {(tai,tbi)|i∈{1,…,(M2)}andtaitbi=+1}; operation |·| stands for set cardinality; and $K=\max_{t_{a},t_{b}\in T}\left|C_{t_{a},t_{b}}\right|$, where *T* is the complete set of ternary training vectors (note that this expression for $C_{t_{a},t_{b}}$ is just a convenient and concise mathematical way to express the set of all pairs of monitors for which both input samples show the same magnitude relationship between the RSSIs of the corresponding monitors, according to the definitions of ternary vectors explained in Section 3.1.2). Note that $|C_{t_{a},t_{b}}|$ is equivalent here to the so-called FreeLoc score in [12], and the chosen formula for $FL_{t_{a},t_{b}}$ is just a sensible way to transform this score into a valid metric in the corresponding ternary vector space.

## 4. Dimensionality Reduction Techniques

Dimensionality reduction techniques try to project a set of high-dimensional vector samples into a space of much lower dimensionality while preserving the relevant global structure information of the data. Aside from the obvious advantages of data compression, one of its main objectives is to provide an easy way to visualize the intrinsic structure of the projected samples. Many different algorithms have been described in the literature to accomplish this task. In this section, we will review some of the most popular, making emphasis on their strengths and potential weaknesses, specifically in the context of processing fingerprinting training data for radio-based location systems.

### 4.1. Principal Component Analysis

Among the general dimensionality reduction techniques, perhaps PCA (Principal Component Analysis) [5] is the most widely known and used. PCA works by finding the linear subspace where the projection of the data minimizes the discrepancy between the distance matrices for every pair of samples in both the input and output spaces. Mathematically, consider a data matrix $X_{N\times D}$, with each row corresponding to an input sample $x_{i}\in R^{D},\forall i=1\ldots N$. Here, *N* is the total number of sample vectors and *D* the number of components of each vector (i.e., D=M, the number of monitors, for the case of raw vectors, or D=(M2)=M*(M−1)2, for the ternary vectors). Importantly, however, the sample mean of each column has been subtracted from each column to obtain a column-wise zero mean on each component.

Under these conditions, the corresponding covariance matrix $\Sigma_{D\times D}$ of the set of vectors can be easily obtained by premultiplying X by its transpose and subsequently eigen-decomposed to obtain a pair of diagonal and orthogonal matrices $\Lambda_{D\times D}$ and $V_{D\times D}$, respectively, such that:

(3) $$\Sigma_{D\times D}=X_{N\times D}^{⊤}X_{N\times D}=V_{D\times D}\Lambda_{D\times D}V_{D\times D}^{⊤}$$

Dimensionality reduction of the original value *D* to a target dimension $D^{\prime }<<D$ works now by simple projection of the input data by post-multiplication of X by the submatrix obtained using only the first $D^{\prime }$ columns of V, i.e.:

(4) $$X_{N\times D^{\prime }}^{\prime }=X_{N\times D}V_{D\times D^{\prime }}$$

Depending on the intrinsic structure and variability of the input data, a variable quantity of information gets lost in this procedure. It can be shown that the explained variance ratio of the projected data $X_{N\times D^{\prime }}^{\prime }$ is bounded by:

(5) $$R=1-\frac{\sum_{i=1\ldots D^{\prime }}\lambda_{i}}{\sum_{i=1\ldots D}\lambda_{i}}$$

where the (positive) $\lambda_{i}$ values are the eigenvalues of the (symmetric and positive semidefinite) matrix Σ, i.e., the elements of the diagonal matrix Λ, sorted in descending order. In particular, if we want to carry out a rapid analysis of the variability in the data, a helpful visualization could be obtained by applying PCA and choosing D’ with a value of two for 2D representation or three for 3D representation. Of course, the quality of the obtained visualization will grow with the value of this explained variance ratio *R*.

Though this simple linear technique certainly does its job well, it also has the drawback that, trying to keep well separated samples, which are very distant in the input space, it tends to entangle the projections of samples, which are not that far apart, thus having the potential negative effect of artificially overlapping nearby clusters. This is the problem that LDA, the technique that we will describe in the next subsection, will try to solve.

### 4.2. Linear Discriminant Analysis

One first solution to the PCA problem is to try to maximize, instead, the separability between classes (rather than the explained variance ratio of the whole population of vectors, as PCA does). This is exactly what LDA (Linear Discriminant Analysis [8]) was designed for. In order to do that, again, the set of input samples $X_{N\times D}$ is going to be linearly projected in a reduced real space $R^{D^{\prime }}$ by multiplying it by an adequate $U_{D\times D^{\prime }}$, just as before, though this time, the U matrix will be chosen such that the separability between classes is maximized, according to the so-called Fisher criterion [18]. This criterion assumes a Gaussian multivariate distribution for each of the *K* classes (i.e., groups of samples obtained in the corresponding *K* zones of interests in the target indoor scenario in our case) and minimizes the area of mutual overlapping between them.

Since, in order to easily optimize the Fisher criterion, LDA assumes that all classes must have the same estimated covariances Σ, a typical previous step is to rescale the data so that their covariance is the identity:

(6) $$X_{r}=\Lambda^{-1/2}V^{⊤}X$$

With $\Sigma =X^{⊤}X=V\Lambda V^{⊤}$ as before. After this normalization, an input data point could be classified by simply finding the estimated class mean that is closest to it, which could be done in turn by first projecting the input using the $U_{D\times D^{\prime }}$ down-projecting matrix resulting from the Fisher optimization criterion.

The main problem of the LDA technique is that a previous labeling of the samples is absolutely needed. This is a clear disadvantage against PCA, which can work with unsupervised (i.e., unlabeled) data. Therefore, LDA is adequate only for supervised learning, and in our domain of application, this means that the zones in which the indoor space is going to be partitioned must be known in advance. This can be a chicken-and-egg problem especially in the early stages of the training, where we first want to know which kind of reliable space partition we could expect to do, an issue that will clearly depend on the structure of the fingerprinting map (i.e., the geographical distribution of the training RSSI samples).

### 4.3. Nonlinear Approaches

PCA and LDA are sometimes limited by their strict linear nature. Extensions to work with more expressive nonlinear manifolds have been developed to try to solve the limitations of linearity, while still working with completely unsupervised data. Among the most widely known are those of kernel-PCA [19], LLE (Locally Linear Embedding [20]) or ISOMAP [21].

In summary, these techniques try to overcome the linear limitations of PCA and LDA by first detecting a presumably continuous, nonlinear low dimensional manifold formed by the data in the high-dimensional space, which is then somehow “unfolded” on the plane or 3D space to easily visualize the results. In order to detect this manifold, a connectivity between each pair of data points must be first evaluated to define a neighborhood graph between the samples.

The problem here is that the estimation of the neighborhood in high-dimensional space can be tricky, especially when the dimension *D* is relatively large with respect to the number of samples *N*. In these conditions, the neighborhood estimation step becomes vulnerable to ’short-circuit’ errors (i.e., non-similar samples getting classified as neighbors) that fail to correctly capture the manifold structure. This is unfortunately much more than merely probable for the inherently sparse case of fingerprinting training databases. The problem tends to get worst, especially in early stages of training, where the dimensionality of the data is typically very high, directly related to the total number of monitors involved, say between a few dozens and a few hundreds in a typical case, while the total amount of obtained vectors during training is kept usually low, as it involves a costly manual data collection process.

This problem tends to make these nonlinear techniques less practical in our field of interest. Still, a recent nonlinear technique completely different in nature has been proposed recently by the machine learning community, which successfully addresses these limitations. We will describe it in the next subsection.

### 4.4. t-Distributed Stochastic Neighbor Embedding

The so-called t-SNE (t-distributed Stochastic Neighbor Embedding) method [10] focuses again on the local structure of the data, trying to extract clustered local groups where similar vectors tend to stand close together, without worrying too much about trying to keep longer distances with the same level of precision. This clearly separates it in spirit from PCA. Instead of assuming a classical Gaussian distribution, just as both PCA and LDA do, t-SNE models distances in the target space using a Student tdistribution, which has much heavier tails than the Gaussian. This way, errors in the long range are penalized much less than in the short range, which is exactly the intended effect.

Though the reader is referred to [10] for details, we give here a brief summary of the t-SNE procedure for completeness. First, t-SNE measures the similarities $p_{ij}$ between every possible pair of training vectors $x_{i}$ and $x_{j}$ in the input (high dimensional) space using a Gaussian probability model, using the following equations:

(7) $$p_{j|i}=\frac{e^{-d_{m}{\left(x_{i},x_{j}\right)}^{2}/2\sigma_{i}^{2}}}{\sum_{k\neq i}e^{-d_{m}{\left(x_{i},x_{k}\right)}^{2}/2\sigma_{i}^{2}}},\;p_{ij}=\frac{p_{j|i}+p_{i|j}}{2}$$

Here, the function $d_{m}\left(\cdot ,\cdot \right)$ refers to the distance between its two input vectors, computed using the desired metric $d_{m}$. In the case of raw vectors, we use the euclidean distance (Equation (1)), i.e., $d_{m}\left(r_{i},r_{j}\right)=EU_{r_{i},r_{j}}$, whereas in the case of ternary vectors, we will use the (more appropriate for passive systems) FreeLoc distance (Equation (2)), i.e., $d_{m}\left(t_{i},t_{j}\right)=FL_{t_{i},t_{j}}$. The variances $\sigma_{i}^{2}$ depend on the so-called perplexity parameter, which is essentially related to the number of neighbors to be considered in every local model centered around each sample. In practice, however, its choice is not critical, since t-SNE is robust to large variations of this parameter. This is in clear contrast with the nonlinear techniques mentioned in the previous subsection (i.e., kernel-PCA, ISOMAP and LLE), which, as has already been explained, can be very sensitive to neighborhood-related parameters.

Then, in a second stage, t-SNE defines a similar probability distribution over pairs in the target (low-dimensional) map, but, crucially, this time using a t Student distribution model:

(8) $$q_{ij}=\frac{\frac{1}{1+\left|\right|y_{i}-y_{j}{\left|\right|}^{2}}}{\sum_{k\neq i}\frac{1}{1+\left|\right|y_{i}-y_{k}{\left|\right|}^{2}}}$$

Here, the target vectors $y_{l}$ refer to the projected versions of the corresponding input vectors $x_{l}$. Observe that, given that these output vectors are no longer defined in the input high-dimensional space, but, rather, on a much lower dimension space for easy visual inspection (typically 2D or 3D), the metric used in this case is always a simple euclidean distance, regardless of the metric used in the first stage.

Finally, the procedure simply minimizes the Kullback–Leibler divergence between the resulting two distributions with respect to the locations of the set of points in the output space $\left\{y_{l}\right\}$, for example using a standard stochastic gradient descent procedure:

(9) $$\min_{\left\{y_{l}\right\}}KL\left(P||Q\right)=\min_{\left\{y_{l}\right\}}\sum_{i}\sum_{j}p_{ij}log\frac{p_{ij}}{q_{ij}}$$

The resulting mapped points $y_{i}\in R^{n}$ would be ready for easy 2D/3D visualization.

## 5. Experimental Study

In the experimental section that follows, we will compare several of the techniques introduced above when working on different conditions and scenarios. More specifically, we will center our discussion on testing PCA, LDA and t-SNE on both raw and ternary vectors in four different datasets. We leave ISOMAP, LLE and kernel-PCA techniques out of our experimental study, mainly for the aforementioned reason that these techniques do not fit well to datasets of relatively small size (up to one or two hundred samples) and high dimensionality, such as the ones that we will use, and that will be described just in the next subsection.

### 5.1. Experimental Scenarios

**Figure 2:**
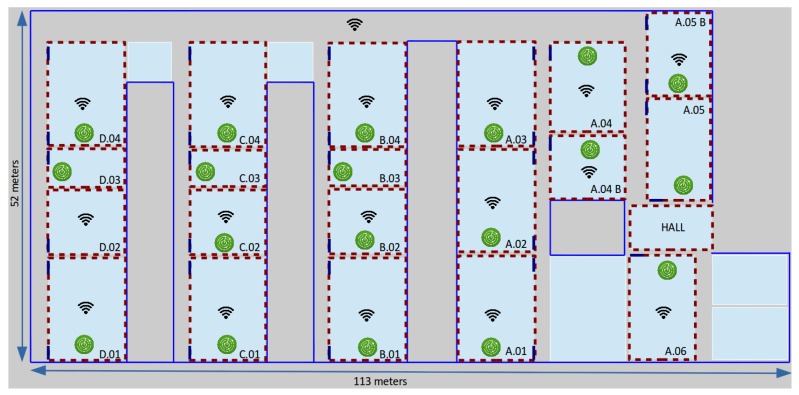
Floor plan of the lecture room building of University of Murcia used for our experiments. The positions of our 19 monitors are shown in green.

We have performed our tests in three buildings with different room distributions, using not only diverse ways to place the monitors, but also different radio technologies, such as WiFi and BLE. Our first (and largest) scenario is a lecture room building of 6000 m${}^{2}$ in the University of Murcia (UMU), Spain. The UMU building has 21 areas of interest, where we have deployed 19 monitors to capture 802.11 traffic transmitted by mobile devices during the operational phase of the localization system (see Figure 2). Since the main purpose of the localization service in this building is to infer room-grain location for large areas (typically large classrooms), the required density of monitors is relatively low: one monitor in each classroom, represented as green circles in the figure, is enough for that purpose. Every classroom, except D.02, is equipped with a teaching computer that we use to install the monitoring software able to capture 802.11 traffic and export it to a central server. The zones of interest, red dotted rectangles in the figure, are twenty classrooms and the main hall, and the existing normal Internet access points, represented as the WiFi symbol in the figure, are distributed throughout the building. This specific experimental environment required about 1 h of training time to cover the whole building, resulting in a final training set of N=226 vectors.

**Figure 3:**
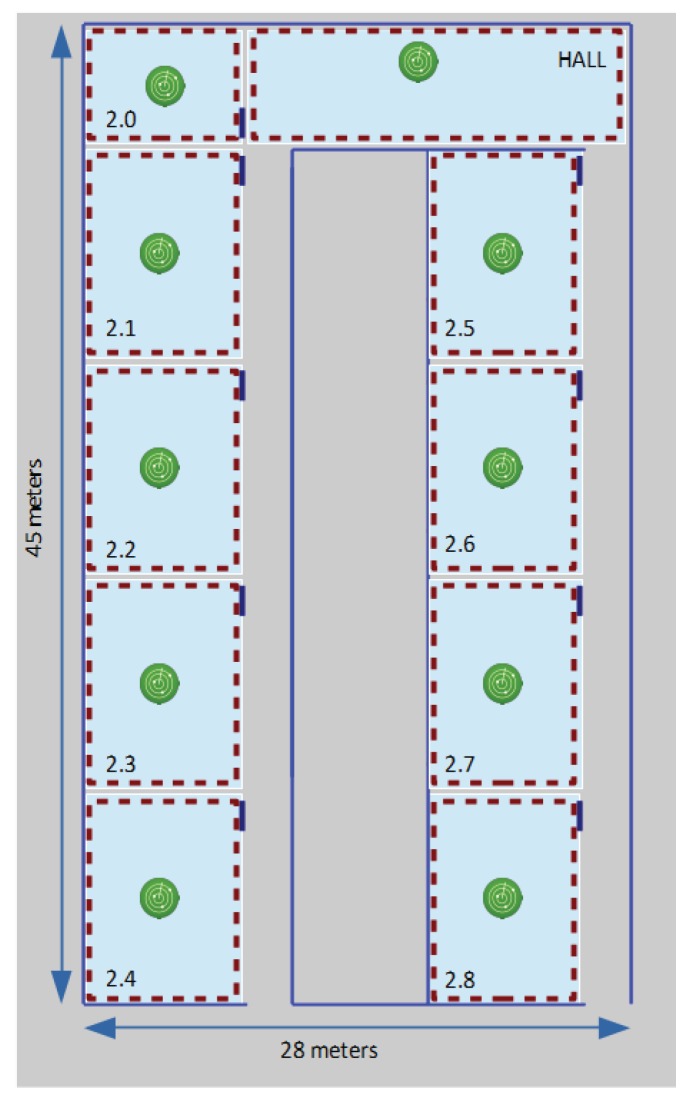
Floor plan of the laboratory building of Computer Science Faculty of University of Murcia used in our experiments. The positions of our 10 monitors are shown in green.

A second scenario is a laboratory building of 1250 m${}^{2}$, located again in our university, more precisely at our Computer Science Faculty (see Figure 3). This UMU laboratory building has 10 areas of interest, where, this time, we implemented a passive localization system based on 10 monitors, now Raspberry Pis endowed with Bluetooth capabilities, capturing BLE traffic transmitted by low cost iBeacon tags, which are in this case the small mobile devices to be localized. In this case, the main goal of the localization service is to infer room-level location information, so, again, one monitor in each laboratory is initially enough for our purposes. BLE monitors are configured to act as iBeacon transmitters during the training phase, in a similar way as we do with WiFi monitors when setting them in access point (AP) mode in 802.11-based systems. The goal is to be able to to measure RSSIs from the training device, just as was explained in Figure 1 for received beacon 802.11 frames. This smaller experimental environment required only about 30 min of training time, resulting in a final training set of N=218 vectors.

Our third scenario is also a lecture room building of 3600 m${}^{2}$ located in the Jaume I Univeristy (UJI) in Castellon (Spain). The UJI lecture building is an experimental scenario extracted from a larger existing database, which was created to compare different indoor localization methodologies based on WiFi fingerprinting [13]. This UJI dataset is being used extensively by the research community (see, for example, [14]), though it is not directly usable in its default form in our case, because it was explicitly created to test active localization systems (i.e., not based on passive monitoring, but on mobile devices willing to be localized and, thus, executing dedicated software for that aim). In any case, taking advantage of the fact that for both active and passive systems the training stage is performed just the same way, i.e., in an active way, just as we explained in Section 2; we will use it as if it would have been generated with a passive, rather than active, localization system in mind. In order to do that, we have had to adapt the dataset to our needs in a way that we will explain in the paragraph that follows.

**Figure 4:**
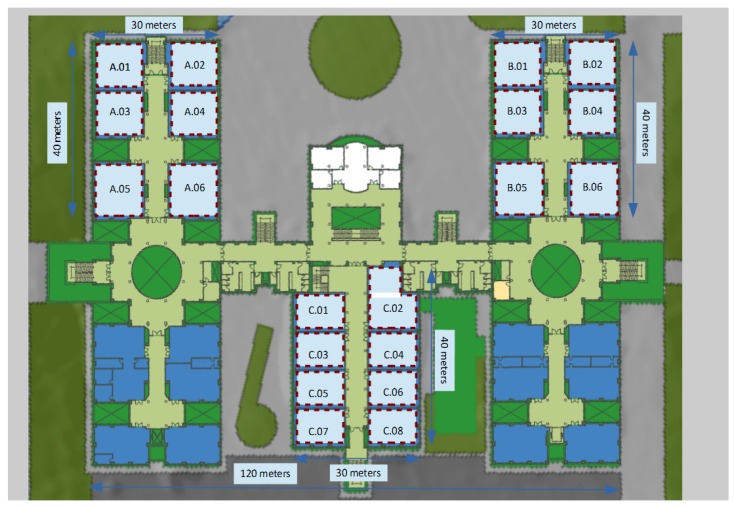
Floor plan of the lecture room building of Jaume I University used for our experiments.

The original UJI dataset covers three buildings of Jaume I University with up to four floors each, and covering a total area of almost 110,000 m${}^{2}$. It was created using more than 20 different users and 25 Android devices and contains more than 20,000 reference records with 529 attributes. These include the WiFi fingerprint of 520 different access points, the geographical coordinates where each sample was taken, as well as other useful data obtained during training (such as device model used, timestamp and the like). In particular, our third scenario uses only the second floor of the second building of this database (see Figure 4). It has 19 areas of interest (Room B.03 is not included in the database) that we also use grouping in only 6 larger zones (close rooms in the same corridor, e.g., A.01, A.02, A.03 and A.04). Filtering the original dataset to get samples only from those zones, the number of records is reduced down to N=239 vectors. In order to create a realistic passive-like experimental scenario, we also filtered the number of APs that are considered: we selected only the 4 most seen APs in each area of interest, resulting in a total of 44 APs for the UJI scenario, which could be a more reasonable number of monitors to deploy for a passive location system in a building of that size. This means that, in order to deploy a passive localization system in that floor and building, we would theoretically have to deploy 44 monitors just in the places where the selected original 44 APs were located.

Our fourth and last scenario is just a subset of the last one, when we consider only the lecture rooms named with the letter “C” in the center of the UJI building, all located around the same corridor, and covering a total surface of approximately 1200 m${}^{2}$. In particular, this last scenario has 8 areas of interest, and the associated filtered training dataset consists of N=79 samples containing values for only 19 APs.

Table 1 summarizes the main features of our four experimental scenarios. Column #RSSIs/vec refers to the average number of active monitors (i.e., number of components with nonzero RSSI values per raw vector). Note that this value is significantly lower for the UMU Laboratories scenario because of the lower power (and thus shorter range) of the BLE technology used for the monitoring.

### 5.2. PCA Results

**Figure 5:**
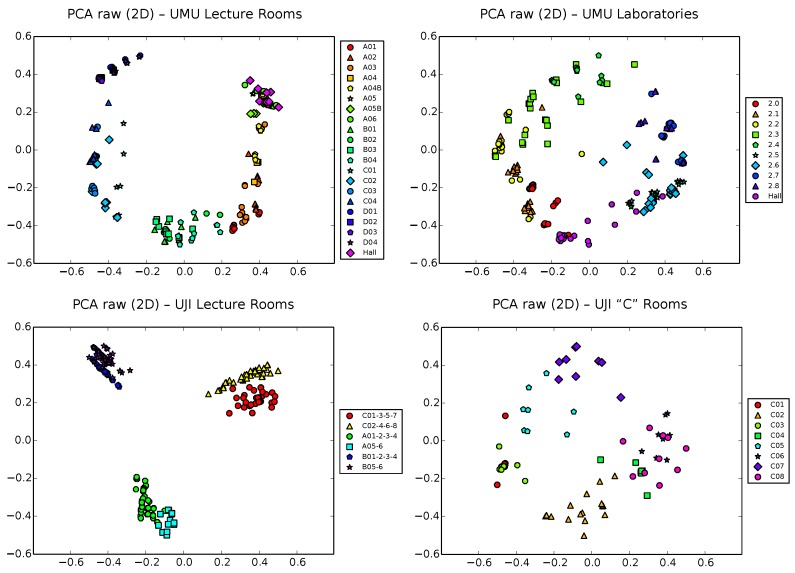
PCA projection of raw samples in 2D space for the UMU1, UMU2, UJI1 and UJI2 datasets.

We start our analysis using classical PCA on the raw vectors of these four training datasets. Figure 5 shows a 2D PCA projection of these raw samples, using different colors and shapes for the different zones of interest of each scenario. Note that colors were chosen to be similar for nearby classrooms/spaces and different marker shapes help in the differentiation of zones. Recall Figure 2, Figure 3 and Figure 4 for the exact situation of zones in each building.

A simple visual inspection of all of the plots in Figure 5 shows that, though clearly distant zones tend to appear well separated, nearby zones clearly tend also to overlap, due to the low sensibility of PCA to separate subsets of more similar samples. This is in general the case for all four datasets, though the situation is slightly more bearable in the UJI1 dataset, where a clear hierarchical structure of three large clusters corresponding to the three wings of the building appears, with two smaller subclusters per corridor, corresponding to the finer level subdivision of two sets of classrooms in each wing. Still, subclusters would be clearly entangled in this dataset if all 19 zones (instead of 6) would have been used to generate the plot.

This is a typical drawback of PCA that, as was discussed in Section 4.1, does not take into account the classes (i.e., zone labels) and tends to capture the global structure of the data, sacrificing precision at a more local scale. The explained variance ratio in two dimensions (Equation (5)) for these PCA projections is approximately 0.7, 0.6, 0.6 and 0.55, for the databases UMU1, UMU2, UJI1 and UJI2, respectively. That is, approximately 30%–45% of information is lost in the 2D projection, taking into account the unexplained variance ratio of the removed dimensions in each case. This is another typical and well-known limitation of PCA.

**Figure 6:**
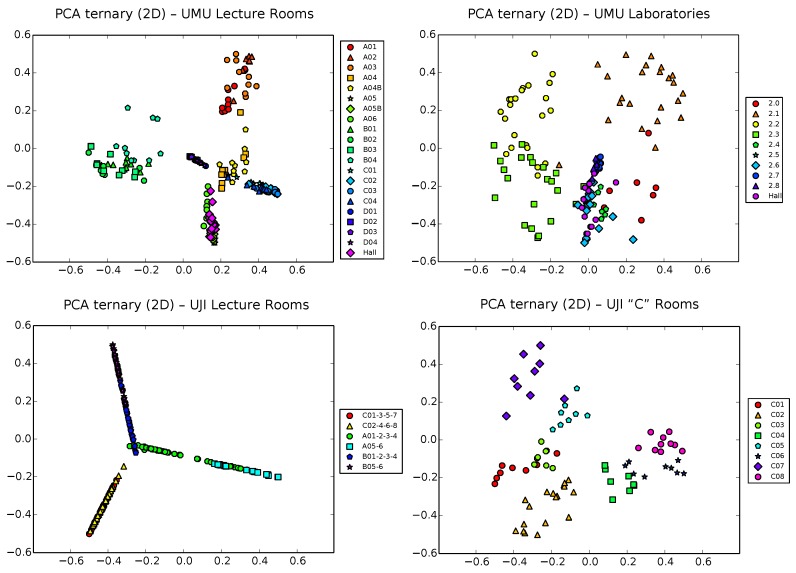
PCA projection of ternary samples in 2D space for the UMU1, UMU2, UJI1 and UJI2 datasets.

Next, we analyze 2D PCA projection of ternary samples (Figure 6). The situation gets slightly better than using raw vectors in some cases (see, for example, the UJI2 dataset on the bottom right part of the figure), though in other cases, PCA manages to separate well only a subset of the classes, at the cost of clearly overlapping others (this is the case, for example, on the UMU2 dataset, top right plot in the figure). A particularly interesting case is that of the UJI1 dataset (bottom left plot), where clusters tend to appear in elongated shapes, while still overlapping. This is probably due to the strong sparse structure of ternary vectors, which, by construction (see Section 3), grow exponentially in size with the input dimension, tending to get filled with zeros when the number of monitors grows (remember that the UJI1 dataset is that with the largest number of monitors, 44). This generates vectors of (442)=44*432=946 dimensions, mostly filled with zeros, except in a few places where the component takes one of the +1/−1 values. These vectors, therefore, are mostly aligned with the input space set of axes, thus tending to be projected in that aligned form.

The explained variance ratios in two dimensions of the ternary vectors are, in this case, of 0.4, 0.3, 0.3 and 0.35 for datasets UMU1, UMU2, UJI1 and UJI2, respectively. This clearly makes for a lesser ratio of covered variability (around only 30%–40%) than in the raw case, due to the inherent higher input dimension of ternary vectors, which results in a higher ratio of lost information when down-projecting to 2D.

In spite of the more expressive power of the ternary vectors, PCA fails again in general to adequately manage the higher input dimension of ternary vectors while preserving separability of zones. This tends to be only acceptable by large zones around corridors, but presents too much overlapping when trying to separate individual classrooms.

### 5.3. LDA Results

**Figure 7:**
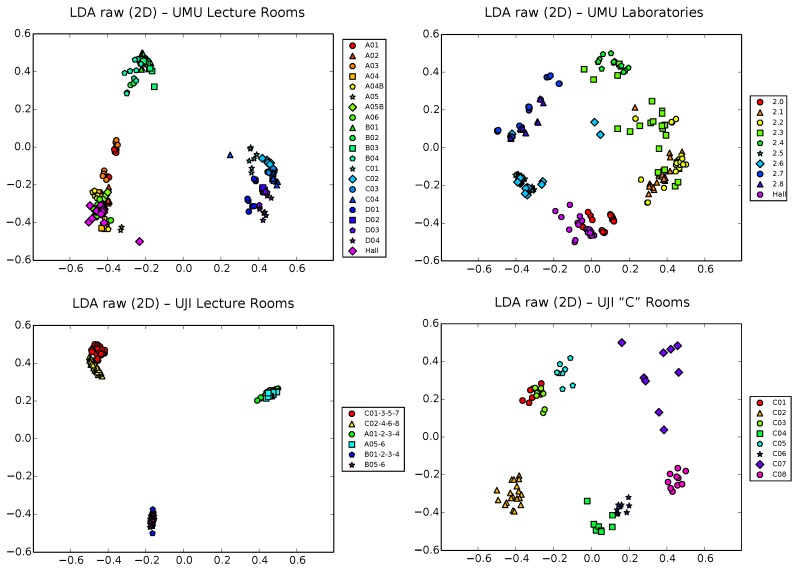
LDA projection of raw samples in 2D space for the UMU1, UMU2, UJI1 and UJI2 datasets.

We proceed now to analyze the results obtained with the LDA technique. First, we will center our study on the raw vectors (see Figure 7). Except for some particular cases (see, for example, UJI2 and, to a lesser extent, UMU2), the results seems not to be much better than those obtained for simple PCA, indicating that the Fisher criterion (Section 4.2) fails here to enhance separability, maybe due to the relatively large number of classes of the different localization scenarios, which tends to need larger input dimensionality to find projection directions acceptable in terms of separation. Remember, also, that the LDA technique needs the zone label of each sample to be able to work, which makes it also less flexible when it comes to obtaining early useful information on a first, fast training survey of the scenario.

**Figure 8:**
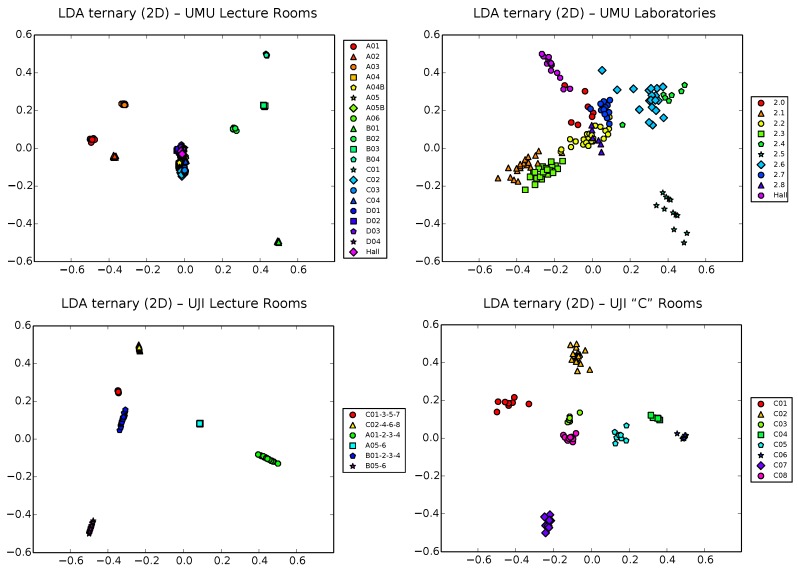
LDA projection of ternary samples in 2D space for the UMU1, UMU2, UJI1 and UJI2 datasets.

The situation certainly gets better in the case of ternary vectors (Figure 8). Though, as we showed in the previous subsection, the exponential growing of the input space can be a problem for PCA, the impact on LDA of this price to pay for using the training to perform passive (and thus necessarily uncalibrated, to allow for heterogeneous devices) localization is, perhaps surprisingly, somehow positive. Except on the UMU1 database (top left plot), where the larger number of zones (21) gets unmanageable for the Fisher criterion optimization, resulting in a clear overlap of most classes in the center of the plot, results for the other three UMU2, UJI1 and UJI2 datasets are visually appealing, with clear separation between clusters, at least when the number of classes is kept low (under 8–10 zones).

Anyway, it is also important to note that one limitation of LDA is that, by definition, it works with the euclidean distance between samples. Though this distance could have also some sense in our ternary vectors (and indeed, it certainly has, as the mentioned results indicate), it is still clearly inferior to the more informative FreeLoc metric shown in Equation (2). This results in good separation of clusters (provided, of course, that the number of classes is not very large), though somehow artificial distances between them, as is clearly observed in the plots of Figure 8. That is, LDA works very well with ternary vectors in terms of separability, though the intrinsic structure of data, i.e., the hierarchical relationship between corridors and classrooms in each corridor, is only partially captured. As we will see shortly, one additional advantage of t-SNE with respect to PCA is that it can work with any user-defined metric, not being limited to the euclidean distance between vectors.

### 5.4. t-SNE Results

**Figure 9:**
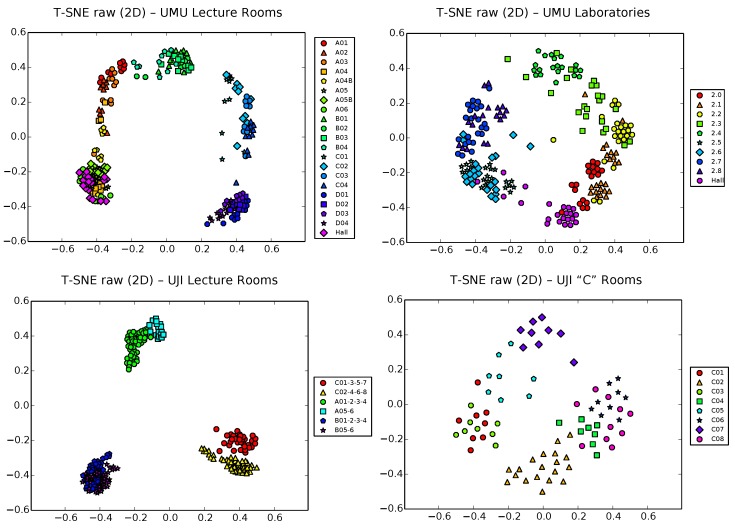
t-distributed Stochastic Neighbor Embedding (t-SNE) projection of raw samples in 2D space for the UMU1, UMU2, UJI1 and UJI2 datasets.

Back to raw vectors, Figure 9 shows now the t-SNE 2D projection of the four datasets. Interestingly, more defined ’first level’ clusters of large zones tend to appear in the harder, 21-zone dataset of UMU1 (top left plot of the figure; observe, for example, the dark blue-violet/lighter blue/green clusters corresponding clearly to the D, C and B classrooms in the corresponding corridors; also, classrooms around the hall and the hall itself form another clear cluster in the lower part of the figure). Moreover, in each of these higher level clusters, ’second level’ clusters start to appear separating classrooms, though some clear cases of overlapping still appear at this finer level. Other more simple cases, such as UJI1 (6 classes) and UJI2 (8 classes) still behave in a similar way, while the UMU2 BLE dataset behaves clearly worst, perhaps due to the lower expressiveness of the BLE raw vectors, with the lowest #RSSIs/vec average ratio (4.70) of all of the datasets.

**Figure 10:**
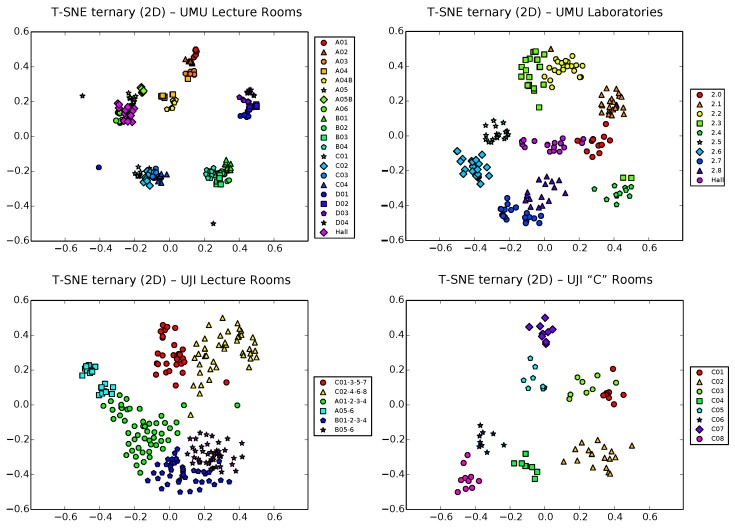
t-SNE projection of ternary samples in 2D space for the UMU1, UMU2, UJI1 and UJI2 datasets.

However, the real potential of t-SNE for fast visual inspection of training data for passive localization systems is shown in Figure 10, where we perform the t-SNE 2D projection of the ternary vector datasets. Taking advantage of the aforementioned capability of t-SNE of working with any metric, the distance used here in the input space is the same used by FreeLoc [12] (Equation (2)).

**Figure 11:**
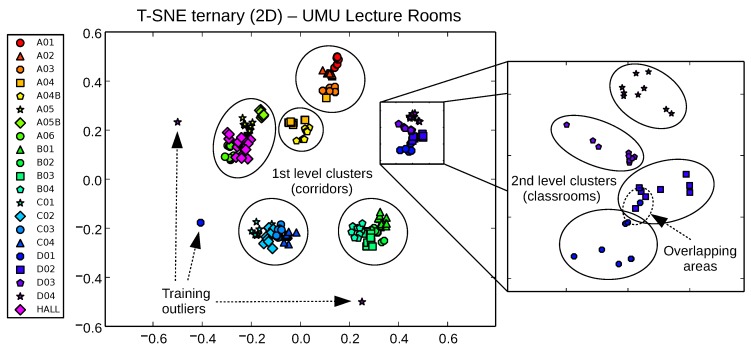
t-SNE projection of ternary samples in 2D space for the UMU1 dataset, with 1st level clusters, 2nd level clusters, training outliers and overlaying areas highlighted.

**Figure 12:**
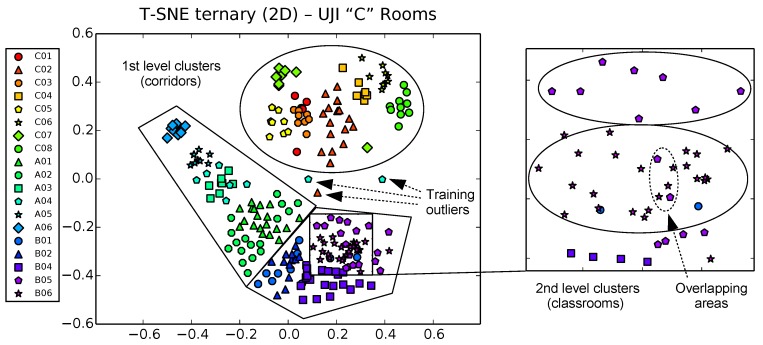
t-SNE projection of ternary samples in 2D space for UJI dataset, using 19 zones instead of 6 (a finer granularity in the zone classification results in a graceful hierarchical distribution of clusters, according to the corresponding topographical relationships between classrooms).

Results are visually better than those obtained by PCA and LDA techniques in terms of both, first, coarse-level clustering and, second, finer level clustering (there is more separability and better distribution between clusters, showing even the hierarchical relationship between corridors and classrooms in each corridor). Classrooms/interest zones appear now clearly clustered by sharing corridors, but taking any of these first level clusters and zooming in, we still see individual classrooms well separated in second level clusters. Areas of potential overlapping can now be attributed to the nature of the radio signals themselves, more than to limitations of the dimensionality reduction procedure. Potentially conflictive samples (outliers due to sporadic signal loss during training) can also be isolated and removed easily by a simple inspection. Figure 11 and Figure 12 detail these nice properties for both the UMU1 (21 zones) and UJI1 (this time using 19 classes instead of 6) harder datasets.

### 5.5. Discussion

Table 2 summarizes the main conclusions of our extensive experimental study. We observe that, in general, t-SNE has a number of advantages with respect to previous alternatives, being the most relevant for our use case:

- It is non-linear in nature and, thus, more expressive than PCA (Principal Component Analysis) and LDA (Linear Discriminant Analysis).
- It is ready to work with unsupervised data and, thus, very flexible to aid in visualization in early stages of training, where the distribution of geographical zones has not been decided yet.
- It can work with any valid metric, not only euclidean, which gives us a enormous flexibility when working with the FreeLoc metric on ternary vectors.
- Finally, it naturally adapts to both dense and sparse input data, better than kernel-PCA, LLE (Locally Linear Embedding) and ISOMAP, which typically require a certain degree of non-sparsity to work reasonably.

On the downside, the technique is certainly computationally more complex, involving a nonlinear optimization process, which, in some cases, must be tuned to avoid possible local minima. In practice, however, in spite of its relative novelty, there are already many efficient and open t-SNE implementations available, a fact that has also contributed to a successful and wide adoption of the technique in many different fields, such as computer vision, speech recognition or natural language processing, among many others [22].

## 6. Cluster Visualization on Scenario Maps

**Figure 13:**
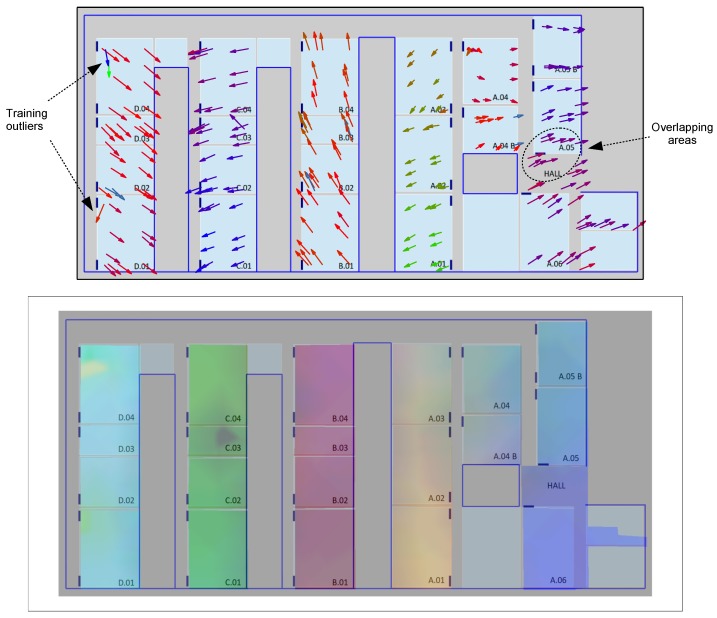
Visualization of t-SNE ternary on UMU Lecture Rooms map (UMU1 dataset). (**Top**) t-SNE arrow map projection. (**Bottom**) t-SNE color map projection.

In addition to the cluster visualization techniques shown in the previous section, we also propose two alternative visual methods based on overlying adequate information of the projected sample points on the respective scenario maps. These alternative visualizations, again based on the versatile t-SNE technique, can further help in a fast and easy diagnosis of potential training problems in a given scenario. In particular, we propose two alternative methods that can result in two different types of representations: an *arrow map* and a *color map*. Both representations are first illustrated in Figure 13 for the UMU1 dataset, by projecting the corresponding ternary vectors dataset in 3D (instead of 2D, as before) using again t-SNE.

Firstly, the arrow map (Figure 13, top) is built by first transforming each resulting output 3D vector in a 2D arrow using its first two coordinates and then using the third coordinate to color the arrow using a continuous palette, which maps values in the [−1.0,+1.0] interval to different colors in the RGB range. Finally, the obtained colored arrows for each of the t-SNE reduced dimension samples are placed directly in the scenario using the (x,y) position, which was adequately geo-tagged during the training process. The result is an appealing geo-positioned visualization technique that might help system designers in rapidly evaluating the quality of the RSSI samples, detecting potentially conflictive zones or even problematic individual samples. For example, the arrow map for the UMU1 dataset shown in the figure marks some training outliers in D.04 and D.01 classrooms and an overlapping area between the hall and the A.05 classroom.

Alternatively, the color map (Figure 13, bottom) tries to rapidly show the degree of separability between particular areas of interest by transforming each t-SNE output 3D vector directly to the RGB color space, plotting again a colored point on the corresponding (x,y) position and using afterwards a simple color diffusion process to smooth the obtained colored map, which is then directly overlaid on the scenario map. As can be rapidly observed in that figure, the color map obtained for the same UMU1 dataset allows one to easily identify the separability between groups of classrooms located at different corridors, as well as potential conflictive training samples in the D.04 and C.03 classrooms.

**Figure 14:**
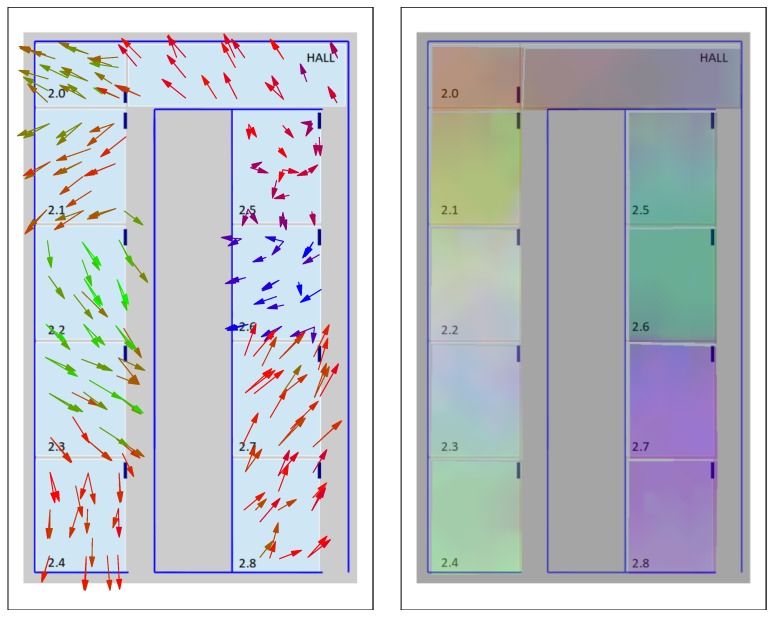
Visualization of t-SNE ternary on UMU Laboratories map (UMU2 dataset). (**Left**) t-SNE arrow map projection. (**Right**) t-SNE color map projection.

**Figure 15:**
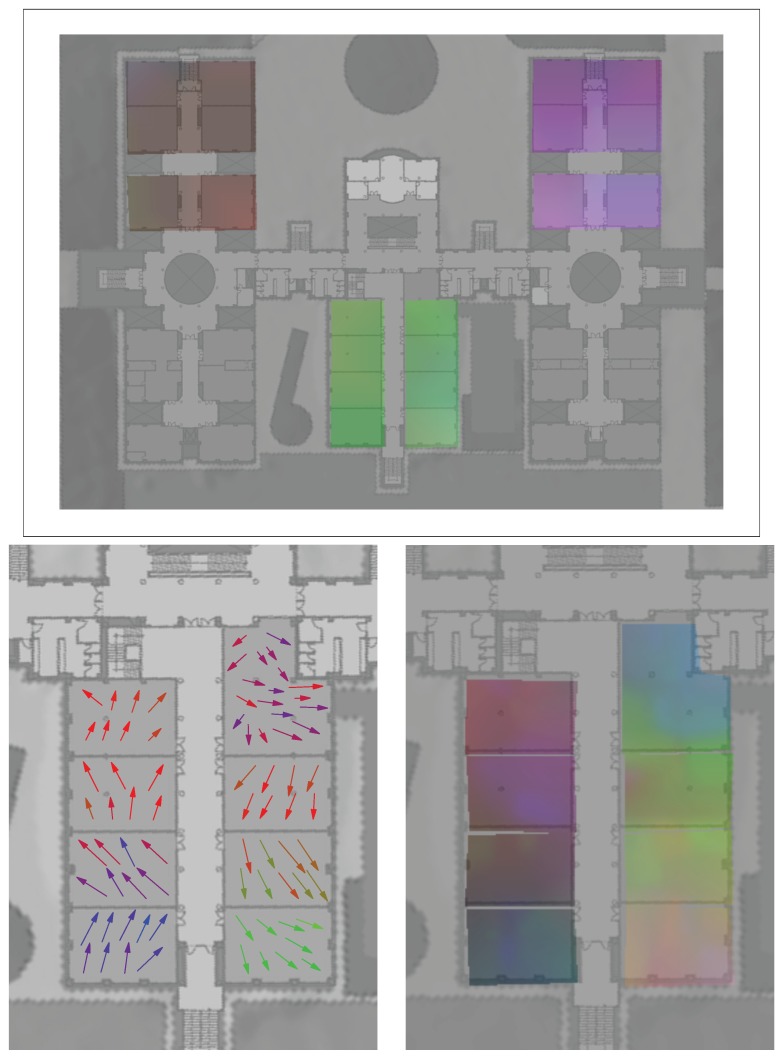
(Top) t-SNE color map projection on UJI lecture rooms (UJI1 dataset). (**Bottom**, **left**) t-SNE arrow map projection for UJI “C” rooms (UJI2 dataset). (**Bottom**, **right**) t-SNE color map projection for UJI “C” rooms (UJI2 dataset). Note: (x,y) positions have been altered manually in this last dataset by moving them to slightly displaced positions, always without going out of each corresponding classroom. This was needed to improve visibility of the obtained color and arrow maps, as the original dataset was geo-tagged only using the center coordinates of each classroom for each subgroup of around 10 samples taken at each individual classroom.

Our t-SNE-based visualization maps could also be used at varying levels of detail, allowing a zoom-in process to provide more detailed visual information to analyze a particular sub-zone of interest in the signal maps. For example, the arrow and color maps shown in Figure 14 for the UMU2 dataset are focused on a smaller area, and clearly show an overlapping area between 2.7 and 2.8 classrooms. A similar analysis can be performed if we focus on the UJI2 dataset (which, as was already discussed in Section 5.1, is in fact a subset of the larger UJI1 dataset, examined at a finer grain level); see Figure 15. Here, the larger UJI1 dataset color map (top figure) shows a clear separation between the three wings of the building around the respective A, B and C corridors, corresponding to three well-defined sets of colors. Then, the arrow and color maps of the reduced (zoomed in) UJI1 dataset (shown on the bottom and right maps of the figure, respectively) clarify the potential separability between the eight classrooms of the south wing of the building, though a certain degree of overlap can still be appreciated between C.01 and C.03 classrooms, for example.

Observe also that, again, any of these t-SNE visualizations is built in a completely unsupervised way, that is without any previous labeling of samples according to zones, since this is not required by t-SNE. Consequently, the proposed visualization can be used to define plausible zone divisions or to suggest changes in the position of the monitors (or adding new ones) when some key zones do not appear clearly separated.

## 7. Conclusions and Future Work

In this paper, we have shown that dimensionality reduction can be an exceptional tool to aid in the deployment cycle of indoor positioning systems based on radio fingerprinting. It can be used to provide fast and intuitive visual feedback about the quality of the resulting radio map for a particular system design, as defined by a tentative zone partition and a corresponding set of monitor positions. We have performed an exhaustive experimental analysis, where several dimensionality reduction techniques have been applied to different datasets. Those datasets do not only differ in the physical structure of their respective scenarios, but also in that they employ different radio technologies, such as 802.11 and BLE.

This thorough analysis has shown that t-SNE provides the best visualization results, especially when working with our proposed ternary vectors and the associated FreeLoc metric, thus correctly addressing the problematic issue of device heterogeneity that typically characterizes passive systems. The t-SNE technique has been shown to be particularly adequate to discover the hierarchical structure of the observations and to detect, in an easy and visual way, overlapping areas or possible outliers. Moreover, the ability of t-SNE to work with non-supervised data can be especially useful to derive a tentative definition of zones of interest that can be used as the starting point of a posterior refinement of the system design.

Additionally, the two different visualization techniques that we have presented (based on arrows and colors overlaid on scenario maps) take advantage of projecting the ternary vectors in 3D in order to provide a quick and straightforward feedback to the operator about the separability of the zones of interest. They are useful tools to define and to rapidly test changes in the number and/or position of the monitors, following the stages of our proposed refinement cycle.

As a statement of direction, we are currently working on using t-SNE and the corresponding geo-tagged down-projected samples as a basis for automatic hierarchical clustering, to further aid in the automation of the zone definition process.

## Figures and Tables

**Table 1:** Main features of the experimental scenarios. vec, vectors.

*Short Name*	*Database*	*Type*	#*Vectors*	#*Monitors*	#*Zones*	#*RSSIs/vec*	*Scenario Size*
**UMU1**	UMU Lecture rooms	WiFi	226	19	21	9.02	6000 m${}^{2}$
**UMU2**	UMU Laboratories	BLE	218	10	10	4.70	1250 m${}^{2}$
**UJI1**	UJI Lecture rooms	WiFi	239	44	6/19	10.99	3600 m${}^{2}$
**UJI2**	UJI “C” Lecture rooms	WiFi	79	19	8	11.33	1200 m${}^{2}$

**Table 2:** Summary of the features of the studied dimensionality reduction techniques.

*Algorithm*	*Type*	*Locality*	*Supported Metrics*	*Supervision*	*Robustness to Sparsity*	*Computational Complexity*
**PCA**	Linear	Low	Euclidean	Not needed	High	Low
**LDA**	Linear	Medium	Euclidean	Needed	High	Low
**kernel-PCA**	Nonlinear	Medium	Any	Not needed	Medium	Medium
**LLE, ISOMAP**	Nonlinear	High	Any	Not needed	Low	Medium
**t-SNE**	Nonlinear	High	Any	Not needed	High	High
